# Exceptional sensitivity of testicular germ cell tumour cell lines to the new anti-cancer agent, temozolomide.

**DOI:** 10.1038/bjc.1995.176

**Published:** 1995-05

**Authors:** M. F. Pera, B. Köberle, J. R. Masters

**Affiliations:** Department of Zoology, Oxford University, UK.

## Abstract

Metastatic testicular germ cell tumours are cured in approximately 85% of patients using cisplatin-based combination chemotherapy. Patients who fail to respond have a poor prognosis, and there is a need for more effective treatments for cisplatin-resistant disease. In this study, it is shown that two of four cell lines derived from human non-seminomatous testicular germ cell tumours are exceptionally sensitive to temozolomide, a new imidazotetrazine which can cross the blood-brain barrier in mice. In addition, three pairs of cisplatin-resistant sublines show little cross-resistance to temozolomide. These data suggest that temozolomide might have activity against non-seminomatous testicular germ cell tumours which have relapsed following cisplatin-containing chemotherapy, and could have a role in the treatment of patients with metastatic lesions in the brain.


British Journal of Cancer (1995) 71, 904–906

© 1995 Stockton Press All rights reserved 0007–0920/95 $12.00

SHORT COMMUNICATION

### Exceptional sensitivity of testicular germ cell tumour cell lines to the new anti-cancer agent, temozolomide

MF Pera[1], B Köberle[2] and JRW Masters[2]

[1]CRC Growth Factors, Department of Zoology, Oxford University, South Parks Road, Oxford OX1 3PS, UK; [2]Institute of Urology and Nephrology, University College London, 3rd Floor Research Laboratories, 67 Riding House Street, London W1P 7PN, UK.

**Summary** Metastatic testicular germ cell tumours are cured in approximately 85% of patients using cisplatin-based combination chemotherapy. Patients who fail to respond have a poor prognosis, and there is a need for more effective treatments for cisplatin-resistant disease. In this study, it is shown that two of four cell lines derived from human non-seminomatous testicular germ cell tumours are exceptionally sensitive to temozolomide, a new imidazotetrazine which can cross the blood–brain barrier in mice. In addition, three pairs of cisplatin-resistant sublines show little cross-resistance to temozolomide. These data suggest that temozolomide might have activity against non-seminomatous testicular germ cell tumours which have relapsed following cisplatin-containing chemotherapy, and could have a role in the treatment of patients with metastatic lesions in the brain.

**Keywords:** testicular germ cell tumour; temozolomide; chemotherapy

Despite the high cure rate obtained with combination chemotherapy of non-seminomatous testicular germ cell tumours, the management of patients with an adverse prognosis at presentation, or of those who fail to respond to first-line chemotherapy, remains a therapeutic challenge (Horwich *et al.*, 1993). Most experimental protocols aimed at these poor-risk groups, which constitute about 15% of the patient population, involve intensification of chemotherapy with agents already known to be active against the disease. It may be that the addition to these regimens of a new drug with a different molecular mechanism of action would have a positive impact on the outcome of treatment.

Cell lines derived from human non-seminomatous testicular germ cell tumours are highly sensitive to cisplatin (Pera *et al.*, 1987*a*; Walker *et al.*, 1987). The pattern of sensitivity observed *in vitro* appears to reflect the clinical response, as testis tumour cells are 3–10 times more sensitive to cisplatin, bleomycin and etoposide than bladder cancer cells, comparing $IC_{50}$ values (Masters *et al.*, 1993). These cell lines are also highly sensitive to another class of DNA-damaging agent: compounds which can alkylate the $O^6$ position of guanine (Walker *et al.*, 1992). The mutagenic consequences of $O^6$ alkylation of guanine are well described. In addition, some mammalian cells are acutely sensitive to the cytotoxic effects of compounds which generate this lesion (reviewed in Roberts, 1978).

Temozolomide, which undergoes chemical decomposition to yield a monofunctional alkylating agent capable of binding to the $O^6$ position of guanine, is a new anti-cancer agent which is less toxic than mitozolomide (Newlands *et al.*, 1992) and which has the ability to cross the blood–brain barrier in mice. We compared the sensitivities to temozolomide of a series of human bladder and testicular germ cell tumour cell lines and three pairs of parent and cisplatin-resistant sublines.

### Materials and methods

The origins of the cell lines used in this study are shown in Table I. All cell lines were maintained routinely under identical conditions as monolayers in 25 $cm^2$ tissue culture flasks (Nunc, Gibco, Paisley, UK) using alpha minimum essential tissue culture medium (αMEM) supplemented with a single batch of 5% heat-inactivated fetal calf serum (Imperial Labs) and 2 mM L-glutamine (Flow, Irvine, UK) in a humidified atmosphere of 5% carbon dioxide in air at 36.5°C. The cells were used over a restricted range of ten passages to minimise changes that might occur during long-term culture.

*Clonogenic assay*

Exponentially growing cells were detached using 0.05% trypsin (Difco 1:250; Difco, London, UK) in an aqueous solution of 0.016% ethylenediamine tetraacetic acid disodium salt (EDTA; BDH Chemicals, Poole, UK) and were plated in 5 cm dishes containing 5 ml of prewarmed and gassed medium at a plating density designed to produce approximately 200 colonies per plate in the untreated controls. Following a 24 h incubation to permit attachment and the resumption of exponential growth, the medium was replaced either with fresh medium alone (in quintuplicate) or containing 0.5% dimethyl sulphoxide (DMSO; Sigma, Poole, UK) or a range of temozolomide (Aston Molecules, UK) concentrations (in triplicate for each concentration).Temozolomide was dissolved in DMSO and the stock solution subsequently added to culture medium. The final volume of DMSO did not exceed 0.5%, a non-toxic concentration. Preliminary experiments identified the range of cytotoxic concentrations, and at least three concentrations which fell in the exponential region of the dose–response curve were selected. After a further 9–15 days' culture, colonies were fixed in methanol (BDH) and stained with 10% Giemsa (BDH). Colonies containing a minimum of 50 cells were counted using a binocular dissecting microscope. The mean colony-forming ability was expressed as a percentage of the untreated controls and computed using least-squares regression analysis on the straight portion of the dose–response plot. The drug was tested against each cell line in at least two further experiments.

### Results

The toxicity of temozolomide to four testis tumour and three bladder cancer cell lines was measured (Figure 1a). Two of

Correspondence: JRW Masters
Received 16 November 1994; revised 1 December 1994; accepted 12 December 1994

Table I Origins and characteristics of the cell lines used in this study

| Cell line designation | Histopathological type | Previous treatment | ATase level[a] (fmol $mg^{-1}$ protein) | Temozolomide mean $IC_{50}$ ($\mu g\ ml^{-1}$) | References |
|---|---|---|---|---|---|
| SuSa | NSTGCT primary | None | 206 | 23.2 | Hogan *et al.* (1977) |
| SuSa-CP | Cisplatin-resistant subline | | 470 | 50.5 | Walker *et al.* (1990) |
| 833K | NSTGCT (abdominal metastasis) | Chemotherapy[b] | ND | 27.6 | Bronson *et al.* (1980) |
| GCT27 | NSTGCT primary | None | 3.3 | 0.54 | Pera *et al.* (1987*b*) |
| GCT27-CP | Cisplatin-resistant subline | | ND | 0.72 | Kelland *et al.* (1992) |
| GCT44 | NSTGCT (lymph node metastasis) | Chemotherapy[c] | ND | 5.8 | Pera *et al.* (1987*b*) |
| RT112 | TCC primary | None | 387 | 24.2 | Masters *et al.* (1986) |
| RT112-CP | Cisplatin-resistant subline | | 301 | 45.9 | Walker *et al.* (1990) |
| MGH-U1 (T24) | TCC recurrence | None | 603 | 24.5 | Bubenik *et al.* (1973) |
| HT1376 | TCC primary | None | 506 | 30.3 | Rasheed *et al.* (1977) |

NSTGCT, non-seminomatous testicular germ cell tumour; TCC, transitional cell carcinoma of the bladder; ND, not done. [a]From Walker *et al.* (1992). [b]Methotrexate, actinomycin D, cyclophosphamide. [c]Cisplatin, etoposide, bleomycin, vinblastine, methotrexate, doxorubicin, actinomycin D, cyclophosphamide.

**Figure 1** Dose-response curves of testis and bladder cancer cell lines to temozolomide. Cells plated at clonal density were exposed to a range of concentrations of temozolomide, and after a further 9–15 days' culture the colony-forming ability of the treated cells was compared with that of the untreated controls. (**a**) Data for four testis: (●, GCT44; ▲, SuSa; ■, 833K; ◆, GCT27) and three bladder (○, RT112; △, MGH-U1; □, HT1376) cancer cell lines. (**b**) Data for three pairs of parent (▲, SuSa; ◆, GCT27; ●, RT112) and cisplatin-resistant sublines (△, SuSa-CP; ◇, GCT27-CP; ○, R112-CP). Survival for each drug dose was estimated from triplicate dishes, and each experiment was repeated at least twice. The error bars show the standard error of the mean of the separate experiments.

the four testis tumour lines, GCT27 and GCT44, were far more sensitive to temozolomide than the other cell lines. GCT27 was derived from a primary tumour in a patient who had not been treated with chemotherapy. In contrast, GCT44 was derived from a metastatic lesion in a patient who had previously been treated and subsequently died of his disease. GCT44 represents an aggressive biological variant of teratoma (Pera *et al.*, 1987*b*). Comparing the temozolomide sensitivity of three of these lines with their cisplatin-resistant sublines, the relative cross-resistance to temozolomide ranged from 1.3 for GCT27 to 2.2 for SuSa (Figure 1b and Table I), comparing the mean concentrations reducing colony-forming ability by 50% ($IC_{50}$). The sublines were derived by continuous *in vitro* exposure to cisplatin, and exhibit a 4- to 6-fold resistance to cisplatin, comparing doses that reduce clonogenic cell survival by 50%.

### Discussion

Two testis tumour cell lines were shown to be exceptionally sensitive to temozolomide. This finding extends our observation that testis tumour cell lines are particularly sensitive to *N*-nitroso-*N*-methylurea (MNU) and mitozolomide (Walker *et al.*, 1992). This earlier study also demonstrated that GCT27 is more sensitive than the other testis tumour lines studied, with $IC_{50}$ values to MNU and mitozolomide of 1.2 and 0.3 $\mu g\ ml^{-1}$, compared with 12.2 and 1.3 $\mu g\ ml^{-1}$ for SuSa (testis) and 56.9 and 4.5 $\mu g\ ml^{-1}$ for RT112 (bladder). The greater sensitivity of GCT27 to temozolomide, mitozolomide and MNU may be related to its low levels of $O^6$-alkylguanine-DNA alkyltransferase activity (see Table I). Low levels of this enzyme may result in higher levels of $O^6$ alkylation, a potentially toxic DNA lesion.

The exceptional sensitivity of the two testis lines to temozolomide is one reason for testing this new drug in the clinic against testis tumours. The major limitation to the successful treatment of these patients is the presence of cisplatin-resistant disease. Therefore, a further rationale for testing this new agent is the observation that in three independent pairs of cell lines there is relatively little change in the sensitivity to temozolomide in the cisplatin-resistant derivatives. A third reason for testing this agent is its clinical activity against brain tumours (Newlands *et al.*, 1992). Brain metastases occur in 8–15% of patients with testicular tumours, almost always associated with relapse at other sites or as a terminal event (Raina *et al.*, 1993). Temozolomide may provide a more effective treatment for testis tumours which have metastasised to the brain.

**Acknowledgements**

Work in our laboratories is supported by the Cancer Research Campaign.

**References**

BRONSON DL, ANDREWS PW, SOLTER D, CERVENKA J, LANGE PH AND FRALEY EE. (1980). Cell line derived from a metastasis of a human testicular germ cell tumour. *Cancer Res.*, **40**, 2500-2506.

BUBENIK J, BARESOVA M, VIKLICKY V, JAKOUBKOVA J, SAINEROVA H AND DONNER J. (1973). Established cell line of urinary bladder carcinoma (T24) containing tumour-specific antigen. *Int. J. Cancer*, **11**, 765-773.

HOGAN B, FELLOUS M, AVNER P AND JACOB F. (1977). Isolation of a human teratoma cell line which expresses F9 antigen. *Nature*, **270**, 515-518.

HORWICH A, WILSON C, CORNES P, GILDERSLEVE J AND DEARNALEY D. (1993). Increasing the dose intensity of chemotherapy in poor-prognosis metastatic non-seminoma. *Eur. Urol.*, **23**, 219-222.

KELLAND LR, MISTRY P, ABEL G, FREIDLOS F, LOH SY, ROBERTS JJ AND HARRAP KR. (1992). Establishment and characterization of an *in vitro* model of acquired resistance to cisplatin in a human testicular nonseminomatous germ cell line. *Cancer Res.*, **52**, 1710-1716.

MASTERS JRW, HEPBURN PJ, WALKER L AND OTHERS. (1986). Tissue culture model of transitional cell carcinoma: characterization of twenty-two human urothelial cell lines. *Cancer Res.*, **46**, 3630-3636.

MASTERS JRW, OSBORNE EJ, WALKER MC AND PARRIS CN. (1993). Hypersensitivity of human testis-tumour cell lines to chemotherapeutic drugs. *Int. J. Cancer*, **53**, 340-346.

NEWLANDS ES, BLACKLEDGE GRP, SLACK JA, RUSTIN GJS AND OTHERS. (1992). Phase I trial of temozolomide (CCRG 81045: M&B 39831: NSC 362856). *Br. J. Cancer*, **65**, 287-291.

PERA MF, FRIEDLOS F, MILLS J AND ROBERTS JJ. (1987*a*). Inherent sensitivity of cultured embryonal carcinoma cells to adducts of cis-diamminedichloroplatinum(II) on DNA. *Cancer Res.*, **47**, 6810-6813.

PERA MF, BLASCO LAFITA MJ AND MILLS J. (1987*b*). Cultured stem-cells from human testicular teratomas: the nature of human embryonal carcinoma and its comparison with two types of yolk-sac carcinoma. *Int. J. Cancer*, **40**, 334-343.

RAINA V, SINGH SP, KABLE N, TANWAR R, RAO K, DAWAR R AND RATH GK. (1993). Brain metastasis as the site of relapse in germ cell tumor of the testis. *Cancer*, **72**, 2182-2185.

RASHEED S, GARDNER MB, RONGEY RW, NELSON-REES WA AND ARNSTEIN P. (1977). Human bladder carcinoma: characterization of two new tumor cell lines and search for tumour viruses. *J. Natl Cancer Inst.*, **58**, 881-890.

ROBERTS JJ. (1978). The repair of DNA modified by cytotoxic, mutagenic and carcinogenic chemicals. *Adv. Radiat. Biol.*, **7**, 211-432.

WALKER MC, PARRIS CN AND MASTERS JRW. (1987). Differential sensitivities of human testicular and bladder tumor cell lines to chemotherapeutic drugs. *J. Natl Cancer Inst.*, **79**, 213-216.

WALKER MC, POVEY S, PARRINGTON JM, RIDDLE PN, KNUECHEL R AND MASTERS JRW. (1990). Development and characterization of cisplatin-resistant human testicular and bladder tumour cell lines. *Eur. J. Cancer*, **26**, 742-747.

WALKER MC, MASTERS JRW AND MARGISON GP. (1992). $O^6$-alkylguanine-DNA alkyltransferase activity and nitrosourea sensitivity in human cancer cell lines. *Br. J. Cancer*, **66**, 840-843.


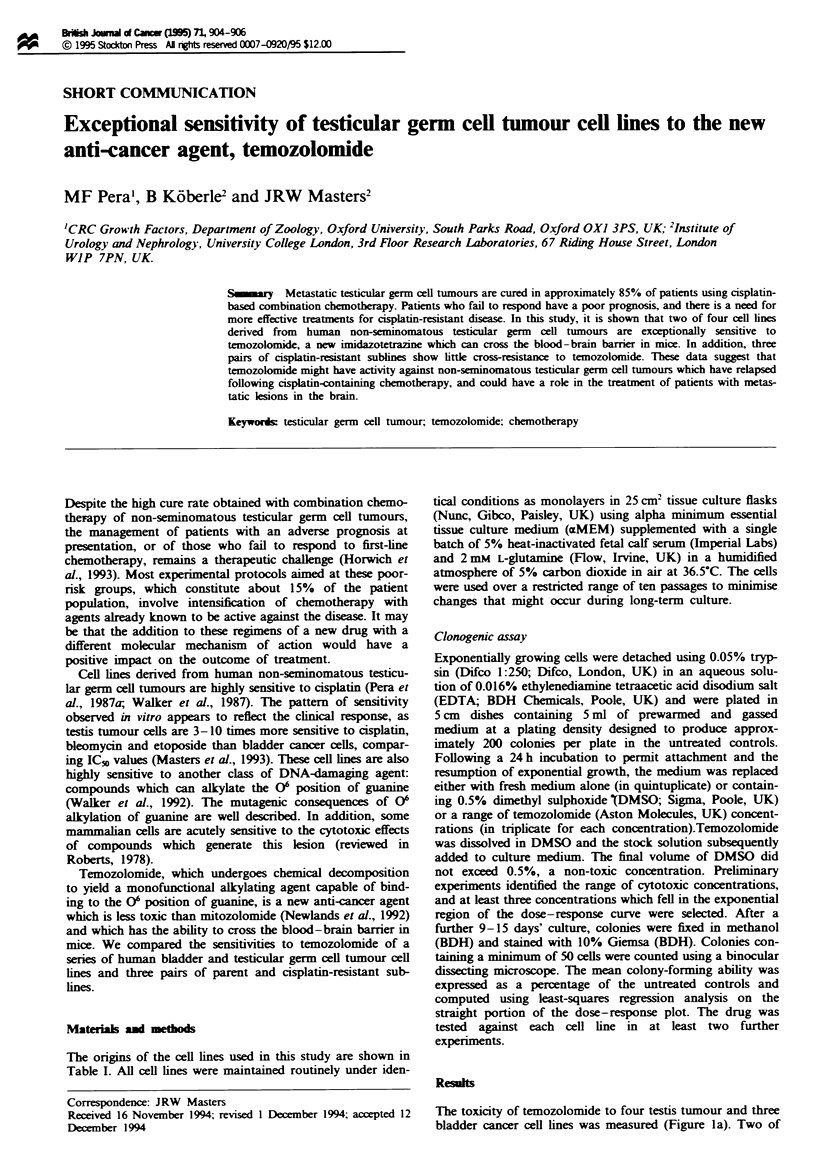

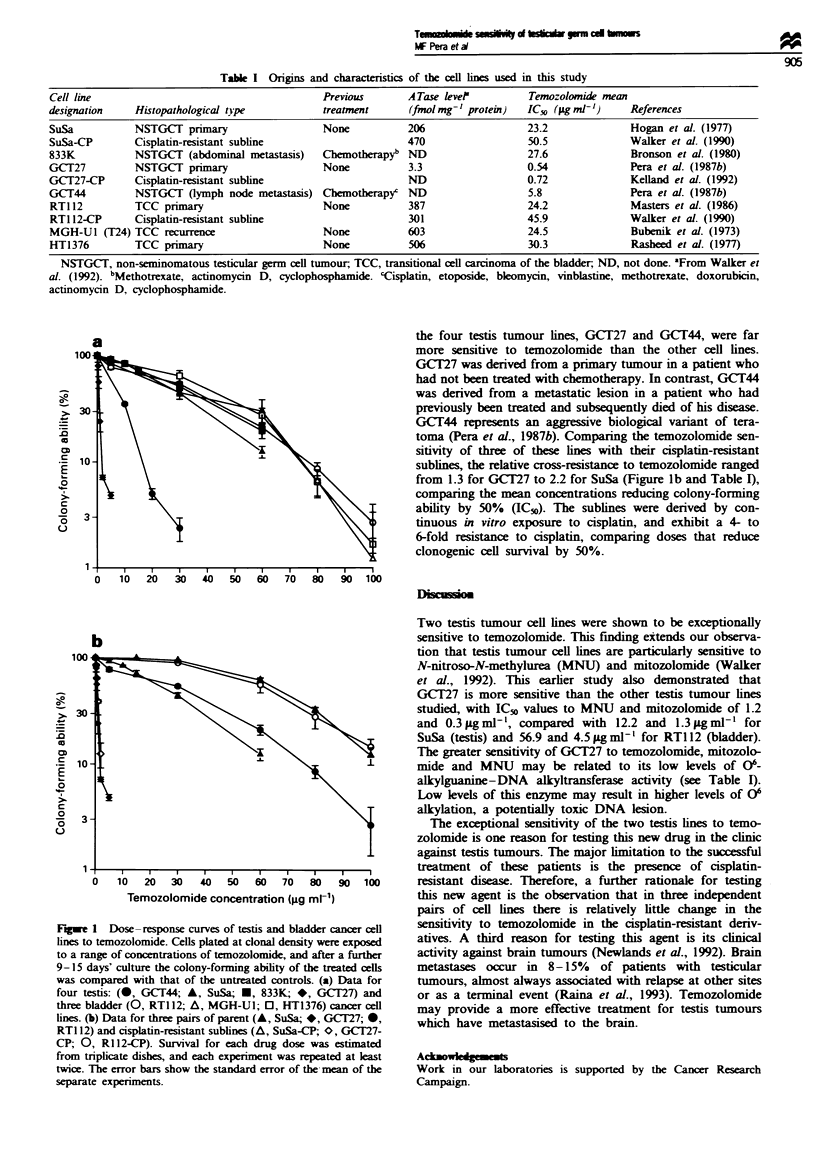

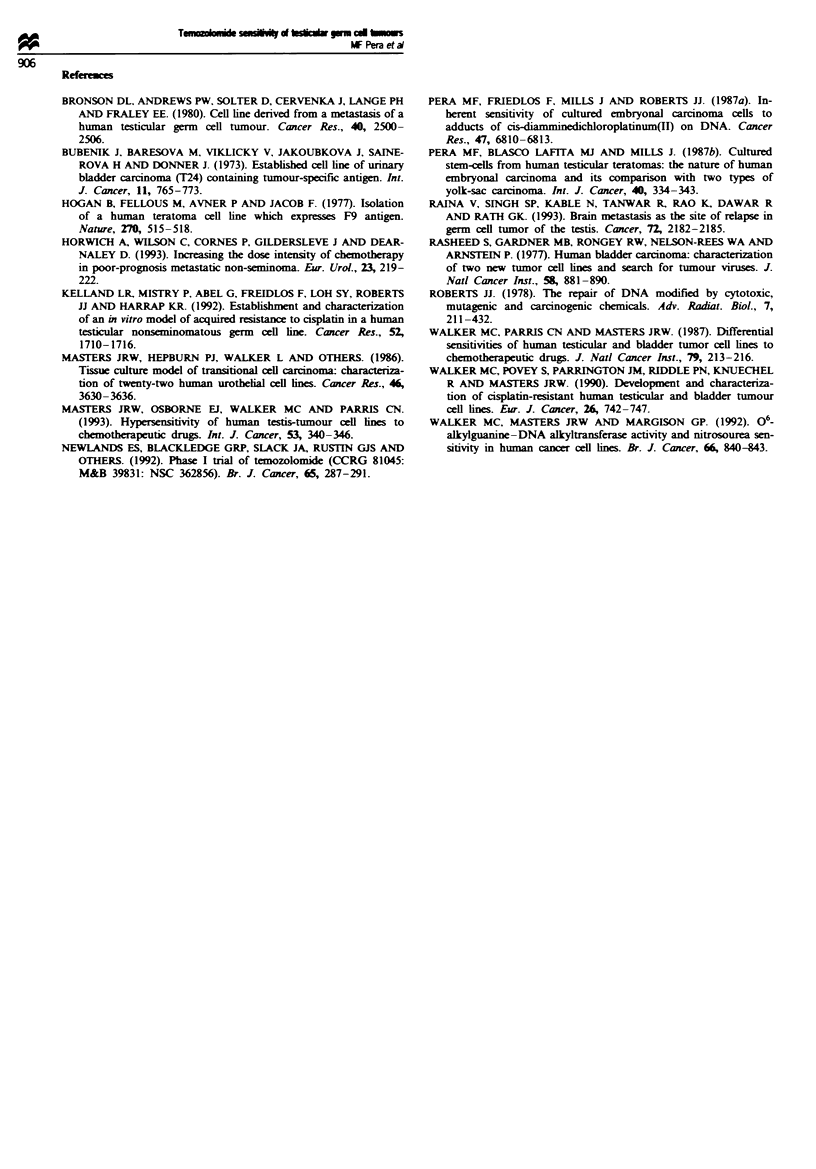


## References

[OCR_00274] Bronson D. L., Andrews P. W., Solter D., Cervenka J., Lange P. H., Fraley E. E. (1980). Cell line derived from a metastasis of a human testicular germ cell tumor.. Cancer Res.

[OCR_00280] Bubeník J., Baresová M., Viklický V., Jakoubková J., Sainerová H., Donner J. (1973). Established cell line of urinary bladder carcinoma (T24) containing tumour-specific antigen.. Int J Cancer.

[OCR_00286] Hogan B., Fellous M., Avner P., Jacob F. (1977). Isolation of a human teratoma cell line which expresses F9 antigen.. Nature.

[OCR_00293] Horwich A., Wilson C., Cornes P., Gildersleve J., Dearnaley D. (1993). Increasing the dose intensity of chemotherapy in poor-prognosis metastatic non-seminoma.. Eur Urol.

[OCR_00299] Kelland L. R., Mistry P., Abel G., Freidlos F., Loh S. Y., Roberts J. J., Harrap K. R. (1992). Establishment and characterization of an in vitro model of acquired resistance to cisplatin in a human testicular nonseminomatous germ cell line.. Cancer Res.

[OCR_00304] Masters J. R., Hepburn P. J., Walker L., Highman W. J., Trejdosiewicz L. K., Povey S., Parkar M., Hill B. T., Riddle P. R., Franks L. M. (1986). Tissue culture model of transitional cell carcinoma: characterization of twenty-two human urothelial cell lines.. Cancer Res.

[OCR_00310] Masters J. R., Osborne E. J., Walker M. C., Parris C. N. (1993). Hypersensitivity of human testis-tumour cell lines to chemotherapeutic drugs.. Int J Cancer.

[OCR_00317] Newlands E. S., Blackledge G. R., Slack J. A., Rustin G. J., Smith D. B., Stuart N. S., Quarterman C. P., Hoffman R., Stevens M. F., Brampton M. H. (1992). Phase I trial of temozolomide (CCRG 81045: M&B 39831: NSC 362856).. Br J Cancer.

[OCR_00328] Pera M. F., Blasco Lafita M. J., Mills J. (1987). Cultured stem-cells from human testicular teratomas: the nature of human embryonal carcinoma, and its comparison with two types of yolk-sac carcinoma.. Int J Cancer.

[OCR_00322] Pera M. F., Friedlos F., Mills J., Roberts J. J. (1987). Inherent sensitivity of cultured human embryonal carcinoma cells to adducts of cis-diamminedichloroplatinum(II) on DNA.. Cancer Res.

[OCR_00332] Raina V., Singh S. P., Kamble N., Tanwar R., Rao K., Dawar R., Rath G. K. (1993). Brain metastasis as the site of relapse in germ cell tumor of testis.. Cancer.

[OCR_00337] Rasheed S., Gardner M. B., Rongey R. W., Nelson-Rees W. A., Arnstein P. (1977). Human bladder carcinoma: characterization of two new tumor cell lines and search for tumor viruses.. J Natl Cancer Inst.

[OCR_00359] Walker M. C., Masters J. R., Margison G. P. (1992). O6-alkylguanine-DNA-alkyltransferase activity and nitrosourea sensitivity in human cancer cell lines.. Br J Cancer.

[OCR_00348] Walker M. C., Parris C. N., Masters J. R. (1987). Differential sensitivities of human testicular and bladder tumor cell lines to chemotherapeutic drugs.. J Natl Cancer Inst.

[OCR_00353] Walker M. C., Povey S., Parrington J. M., Riddle P. N., Knuechel R., Masters J. R. (1990). Development and characterization of cisplatin-resistant human testicular and bladder tumour cell lines.. Eur J Cancer.

